# Stimulating effect of normal-dosing of fibrates on cell proliferation: word of warning

**DOI:** 10.1186/s12944-016-0335-z

**Published:** 2016-09-22

**Authors:** Katerina Cizkova, Jana Steigerova, Jan Gursky, Jiri Ehrmann

**Affiliations:** 1 Department of Histology and Embryology, Faculty of Medicine and Dentistry, Palacky University Olomouc, Hnevotinska 3, 775 15 Olomouc, Czech Republic; 2 Department of Clinical and Molecular Pathology & Laboratory of Molecular Pathology, Faculty of Medicine and Dentistry, Palacky University, Hnevotinska 3, 775 15 Olomouc, Czech Republic; 3 Institute of Molecular and Translation Medicine, Faculty of Medicine and Dentistry, Palacky University, Hnevotinska 5, 775 15 Olomouc, Czech Republic

**Keywords:** Peroxisome proliferator-activated receptor α, Hypolipidemic drugs, Proliferation, CYP Epoxygenases, Epoxyeicosatrienoic acids, Cancer

## Abstract

**Background:**

Fibrates are widely used hypolipidemic drugs, which serve as ligand of peroxisome proliferator-activated receptor α (PPARα). Recently, they have also been considered as potential anticancer agents. We studied effect of fibrates treatment on cell proliferation, expression of CYP2J2 and concomitant changes in expression of cell cycle regulatory proteins in three different human cell lines: HEK293, HepG2, and HT-29.

**Methods:**

We used WST-1 viability test, western blot and immunocytochemistry for detection of proteins of interests and analysis of cell cycle.

**Results:**

Our results showed that at lower concentrations of all tested fibrates, viability of all tested cell lines is increased, whereas at higher concentrations, repression is apparent. Unfortunately, the viability of tested cells is predominantly increased in a range of concentration which is reached in patient plasma. This phenomenon is accompanyed by elevation of CYP2J2, increased number of cyclin E-positive cells and decreased number of Cdc25A-positive cells in all tested cell lines, and elevated cyclin A expression in HepG2 and HT-29. These changes are concentration-dependent. We suppose that increased level of CYP2J2 could explain enhanced cell proliferation in lower concentration of fibrates.

**Conclusion:**

Based on our results, we suggested there is no anti-cancer effect of fibrates in tested carcinoma cell lines.

**Electronic supplementary material:**

The online version of this article (doi:10.1186/s12944-016-0335-z) contains supplementary material, which is available to authorized users.

## Background

Fibrates are well-known ligands of peroxisome proliferator-activated receptor α (PPARα) which are clinically widely used drugs for treatment of dislipidemias. PPARα is ligand-activated transcription factor. After ligand binding, histon deacetylase (HDAC) co-repressors are released and PPARs heterodimerize with retinoid X receptor (RXR). After that, RNA polymerase II and co-activators with histone acetyl transferase (HAT) activity are recruited to this complex. The complex binds to peroxisome proliferator response elements (PPREs) in target genes and transcription of target genes is increased. PPARs can lead to downregulation of gene expression which occurs by interfering with other proteins and transcription factors through a trans-repression mechanism [1]. The transcriptional activity of PPARs can be also affected by crosstalk between phosphorylation and dephosphorylation [2].

PPARα is highly expressed in tissues with active fatty acid metabolism, such as the liver, heart muscle, intestine and kidney [3]. PPARα acts as lipid sensor and play important role in regulation of nutrient metabolism and energy homeostasis. Beside that, it is involved in regulation of inflammation and xenobiotic metabolism [4].

PPARα is activated by various endogenous and exogenous ligands, for example fatty acids, eicosanoids, phtalates, etc. Recently, fibrates have been also considered as potential anticancer agents with relatively low toxicity. Despite this fact, the role of PPARα in cancerogenesis is controversial. There are a lot of studies describing their potential for cancer treatment and chemoprevention [5–11]. On the other hand, Suchanek et al. [12], descibed an increase in proliferation in human breast carcinoma cell lines after PPARα ligands treatment. Thus, the exact role of PPARα and its ligands in cancer remains unclear. It has been described a lot of effects of PPARα ligands on tumours cells and tumours (see Additional file 1) [3, 4, 10, 13–16].

As mentioned above, PPARα plays a role in the regulation of xenobiotic metabolism enzymes. Beside other xenobiotic enzymes, PPARα plays role in regulation of cytochrome P450 (CYPs) enzymes called CYP epoxygenases [4]. These enzymes, mainly CYP2C and CYP2J, convert arachidonic acid to epoxieicosatrienoic acids (EETs) which have generaly cytoprotective effects [16]. The main organs involved in the absorption, distribution, metabolism, and elimination of xenobiotics are liver, intestines, and kidney. Therefore three different human cell lines derived from these organs has been used in this study: HEK293 (human embryonic kidney) and two carcinoma-derivated cell lines HepG2 (hepatocellular carcinoma) and HT-29 (human colorectal adnocarcinoma). We investigated the influence of fibrates on viability of these cell lines and consequent changes in expression of CYP2J2 and cycle regulatory proteins. According to our best knowledge, this is the first study which demonstrate changes in CYP2J2 protein expression in human cell lines after treatment by different concentrations of fibrates.

## Methods

### Cell culture

HEK293 and HepG2 cells were routinely cultured in DMEM (HyClone, SH 30249.01). HT-29 cells were cultured in RPMI-1640(Sigma Aldrich, R5866). Both DMEM and RPMI media were supplemented with 10 % FBS (HyClone, cat. no. SV30180.02) and penicilin (100 U/ml), streptomycin (100 mg/l). Cells were incubated at 37 °C and 5 % CO_2_. Cells were passaged twice per week. All cell lines were obtained from the American Type Culture Collection. The cell lines authentication via STR profiles has been performed by Department of clinical genetics, Palacky University, Olomouc.

### Proliferation assay

We used 4 different PPARα ligands (fibrates): fenofibrate (Cayman, cat. no. 10005368), bezafibrate (Cayman, cat. no. 10009145), gemfibrozil (Sigma-Aldrich, cat. no. G9518), WY-14643 (Sigma-Aldrich, cat. no. C7081) to investigate their effect on proliferation of HEK293, HepG2, and HT-29 cell lines. Fenofibrate, bezafibrate, gemfibrozil, and WY-14643 were solved in dimethyl sulfoxid to stock solution at concentration 10 mM.

Cells were plated in 96-well plates at density of 5000 cells/ well for HEK293 and 10000 cells/well for HepG2 and HT-29 in growth medium. Cells were incubated overnight and then different ligands of PPARα in different concentrations were added. Final volume of the growth media was 100 μl/well. Cells were incubated with ligands for 72 h at 37 °C and 5 % CO_2_. Moreover, cells were incubated in two different concentrations of DMSO (0.1 and 1 %) to confirm that there is no significant influence of DMSO on cell viability.

To quantify the cell viability, 10 μl of WST-1 reagent per well (Roche, cat. no. 11 644 807 001) was added into the growth media. Cells were incubated 90 min at 37 °C and 5 % CO_2_ before the measurement of absorbance at 450 nm with microplate reader Power Wave XS (Bio-Tek).

### Western blot analysis

HEK293, HepG2 and HT-29 cells were incubated for 72 h in presence of all tested fibrates in concentrations with maximal viability and IC10 (obtained by WST-1 viability test, see Result section). Samples were separed by SDS-PAGE in 10 % stacking gel. Then, proteins were transfered to Amesham Hybond-ECL membrane (GE Healthcare). Mouse monoclonal antibody against CYP2J2 (NBP2-01178, NovusBiologicals) was used. As endogenous control, GAPDH expression was used. Detection was performed with mouse monoclonal primary antibody (DAKO). The incubation took overnight. Next day, membranes were washed in PBS Tween 20 buffer and then incubated with secondary antibody (7076, Cell Signalling) for 1 h in room temperature and washed. Detection was accomplished by SuperSignal West Femto Maximum Sensitivity Substrate (34095, Thermo Scientific) and membranes were exposed to Biomax Light Film (Kodak). The films were scanned by densitometer GS-700 (BioRad). Relative protein expression was evaluated by measuring optical density (OD) by ImageJ software (National Institute of Health, USA).

### Immunocytochemistry

To confirm ratio of proliferating cells (by Ki-67) after the treatment by clinically used fibrates (fenofibrate, bezafibrate and gemfibrozil), presence and localization of PPARα receptor and estimate changes in expression of cell cycle regulators cyclin E, cyclin A, and Cdc25A in control and fibrate-treated cell lines, we used two-step immunocytochemical method. The same staining was performed for comfirmation of p53 in tested cell lines. These protein have been chosen based on our preliminary experiments (data not shown).

Cells were treated by those concentrations of PPARα ligands in which WST-1 test showed maximal viability (see Result section) for 72 h. Moreover, based on the results, cell lines were treated also IC10 for detection of chosen antigens. Cells were seated in concentration of 128 000 cells per 1 ml in drop of media on the sterile slides. After cell adhesion (6 h minimum) the fresh media was added and cells were incubated overnight. Next day, cells were treated by PPARα ligands in appropriate concentrations for 72 h. As a control, the cells treated by 0.1 % DMSO were used. Then, slides were washed by PBS buffer,fixed for 10 min in cold methanol:aceton (1:1) solution, air-dried and storage at −20 °C.

Ki-67 by mouse monoclonal antibody (DAKO, N1633) at dilution 1:200, PPARα by rabbit polyclonal antibody (Abcam, ab8934) at dilution 1:50, cyclin E by mouse monoclonal antibody (Santa Cruz, sc-247) at dilution 1:50, cyclin A by rabbit polyclonal antibody (Santa cruz, sc-751) at dilution 1:100, Cdc25A by mouse monoclonal antibody (Santa Cruz, sc-56264) were detected. The appropriate dilution of the antibodies was specified by staining positive controls recommended by manufacturers in datasheets. Antibodies were diluted in Dako REAL™ Antibody Diluent (DAKO). Western blot and immunocytochemistry evaluation data for rabbit polyclonal antibodies are provided by manufacturers.

Before staining, the samples were hydrated by tap water wash for 5 min. To block endogenous peroxidase activity, slides were incubated with 0.3 % H_2_O_2_ for 15 min. Then, heat antigen retrieval was performed. For blocking non-specific background staining, the samples were incubated with Protein Block (DAKO) for 10 min at room temperature. The next step was an incubation of slides with primary antibodies for 1 h at room temperature. The detection of proteins was performed by EnVision™ Detection System, Peroxidase/DAB, Rabbit/Mouse (DAKO). Tris buffer (pH 7.6) was used for washing between different steps. Nuclei of all samples were counterstained with hematoxylin and washed in tap water. Then, samples were dehydrated by passage through ethanol, aceton, and xylen washes and coverslipped.

### Data analysis

Results of proliferation assay (IC50, IC10 and concentration with maximal viability) are shown in Table 1 as an average ± standard deviation (SD) (*n* = 3). Results of cell viability assay were evaluated by Student’s *t*-test at the *p* < 0.05 level of significance. All calculations were performed using MS Excel 2010. Statistically significant results are marked with asterisk (*).

**Table 1:** Maximal viability concentration, IC10, and IC50 of fenofibrate, bezafibrate, and gemfibrozil in tested cell lines

	PPARα ligand	max. viability		IC10 (μM)	IC50 (μM)
		concentration (μM)	relative viability (% of control)		
HEK293	fenofibtrate	1.2 ± 0.8	147.0 ± 19.9	2.5 ± 0.4	12.0 ± 1.8
	bezafibrate	22.5 ± 5.0	129.0 ± 5.1	92.2 ± 25.2	175.3 ± 12.6
	gemfibrozil	27.5 ± 9.6	137.0 ± 25.6	93.1 ± 30.9	180.4 ± 18.9
	WY-14643	10.0 ± 0.0	139.0 ± 8.2	82.4 ± 20.2	141.0 ± 3.8
HepG2	fenofibtrate	20.0 ± 0.0	117.0 ± 7.0	76.1 ± 4.7	188.0 ± 8.5
	bezafibrate	23.3 ± 23.1	139.0 ± 42.6	200.0 ± 8.8	332.3 ± 16.1
	gemfibrozil	10.0 ± 0.0	127.0 ± 10.4	251.3 ± 25.7	350.0 ± 13.3
	WY-14643	76.7 ± 5.8	173.0 ± 20.0	121.9 ± 26.2	265.1 ± 20.1
HT-29	fenofibtrate	13.3 ± 5.8	137.0 ± 10.3	205.7 ± 4.0	327.0 ± 38.0
	bezafibrate	10.0 ± 0.0	141.0 ± 14.4	201.1 ± 27.7	268.8 ± 27.1
	gemfibrozil	76.7 ± 5.8	145.0 ± 14.4	191.6 ± 37.6	261.2 ± 36.8
	WY-14643	63.3 ± 11.5	147.0 ± 21.3	184.0 ± 20.8	263.5 ± 15.3

Table presents summary of detected concentration which promotes viability of the cells (max. viability - concentration) and relative viability of the cells reached in these concentration (max. viability - relative viability as a % of viability of the control cells). IC10 and IC50 concentrations of tested fibrates for estimated cell lines are also displayed. Data are presented as average concentration of fibrate ± SD (*n* = 3)

Prior to evaluation of immunohistochemical samples, the samples were coded to minimize observer bias. Immunocytochemical samples stained for Ki-67, cyclin E, cyclin A, and Cdc25A were evaluated as % of positive cells in 3 different fields of vision, magn. 100×. The average % of positive cells were calculated for both, control and treated cells. The evaluation of PPARα staining was based on the fact that activation of the receptor leads to incerased nuclear positivity. The samples were evaluated as % of nuclear positive cells in 3 different fields of vision, magn. 100×. The whole set of experiment was performed three-times.

The results obtained for Ki-67, cyclin E, cyclin A, and Cdc25A are shown as average % of positive cells ± SD (*n* = 3). Moreover, the fold change for all treated samples in comparison to control cells were counted as following:

$$ fold\  change = \frac{average\ \%\  of\  positive\  cells\  in\  treated\  sample\ }{average\ \%\  of\  positive\  cells\  in\  control\  sample} $$

These results are shown in graphs as fold change on log 2 scale. Data were also evaluated by Student’s *t*-test at the *p* < 0.05 level of significance. All calculations were performed using MS Excel 2010. Statistically significant results are marked with asterisk (*).

### Cell cycle analysis

Cells were plated at 6 cm cultivation dish at density of 2x10^5^ cells/dish for HEK2593 and 4x10^5^ cells/dish for HepG2, and HT-29 in appropriate growth medium. Cells were incubated overnight and then treated by fenofibrate, bezafibrate, and gemfibrozil in maximal viability concentrations and IC10. Cells were incubated 24 h in presence of tested ligands (37 °C, 5 % CO_2_). As a control, the cells treated by 0.1 % DMSO were used. Following trypsinization, cells were washed in PBS and centrifuged at 2000 rpm for 10 min. Then, supernatants were removed and cells were fixed by ice cold 70 % etanol for 30 min on ice. Fixed cells were washed by 0.1 % sodium citrate and centrifuged at 2500 rpm for 5 min. Supernatants were removed and propidium iodide solution (100 μg/1 ml) was added (600 μl of PI per sample), cells were incubated 15 min at 37 °C. Then, 400 μl of RNase per sample were added and incubated again for 15 min at 37 °C. Before measurement, cells were left at least for 30 min on ice. Cells were analyzed for cell cycle using flow cytometer BD FACSVerse (BD, USA) which provided histograms to evaluate cell cycle distribution.

## Results

### Viability of HEK293, HepG2, and HT-29 cells after treatment by various PPARα ligands

We investigated effects of 4 different PPARα ligands (fenofibrate, bezafibrate, gemfibrozil, WY-14643) on viability of HEK293, HepG2 and HT-29 cell lines. We determined IC50 and IC10 concentrations for all investigated ligands. Moreover, at lower concentrations of the ligands, an increase of proliferation in all tested cell lines was apparent for all tested ligands. Hence, we determinated concentrations of ligands when cell viability was maximal. Relative viability of cells in a concentration of maximal viability vary from 117 to 173 % of control. All these results (IC50, IC10, and maximal viability concentration) for all three cell lines are summarized in Table 1. To confirm that DMSO treatmen does not significantly affect cell viability, cells were treated by 0.1 and 1 % DMSO. For results, see Additional file 2.

**Fig. 1:**
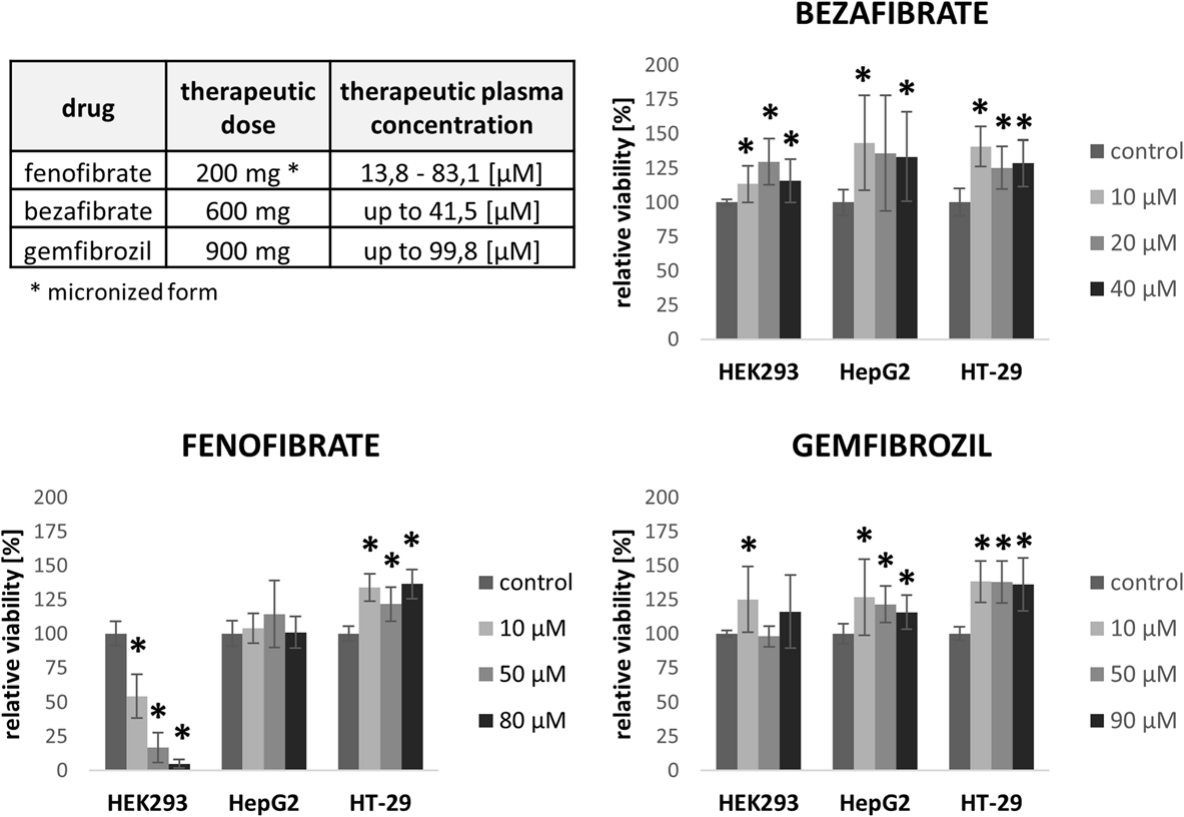
Viability of cells in concentration range which is reached in patients plasma after therapeutic dose of fibrates. Viability of tested cell lines is predominantly incerased after treatment by fibrates in a range of concentration which is reached in patient plasma after normal dosing. * Statistically different from control value at *p* < 0.05

Because of fenofibrate, bezafibrate, and gemfibrozil widen clinical utilization, we defined cell viability at 3 different concentrations in range of therapeutic plasma concentrations of these drugs which is reached in patient plasma after normal dosing of these drugs. The therapeutic doses and plasma concentrations of these drugs and relative viability of tested cell lines after treatment are summarized in Fig. 1. All three ligands, with exception of fenofibrate in HEK293 cell lines, showed an increase of proliferation in these ranges of concentrations in comparison to control cells. Statistically significant results are marked by * (*p* < 0.05), for *P* values, see Additional file 3.

**Fig. 2:**
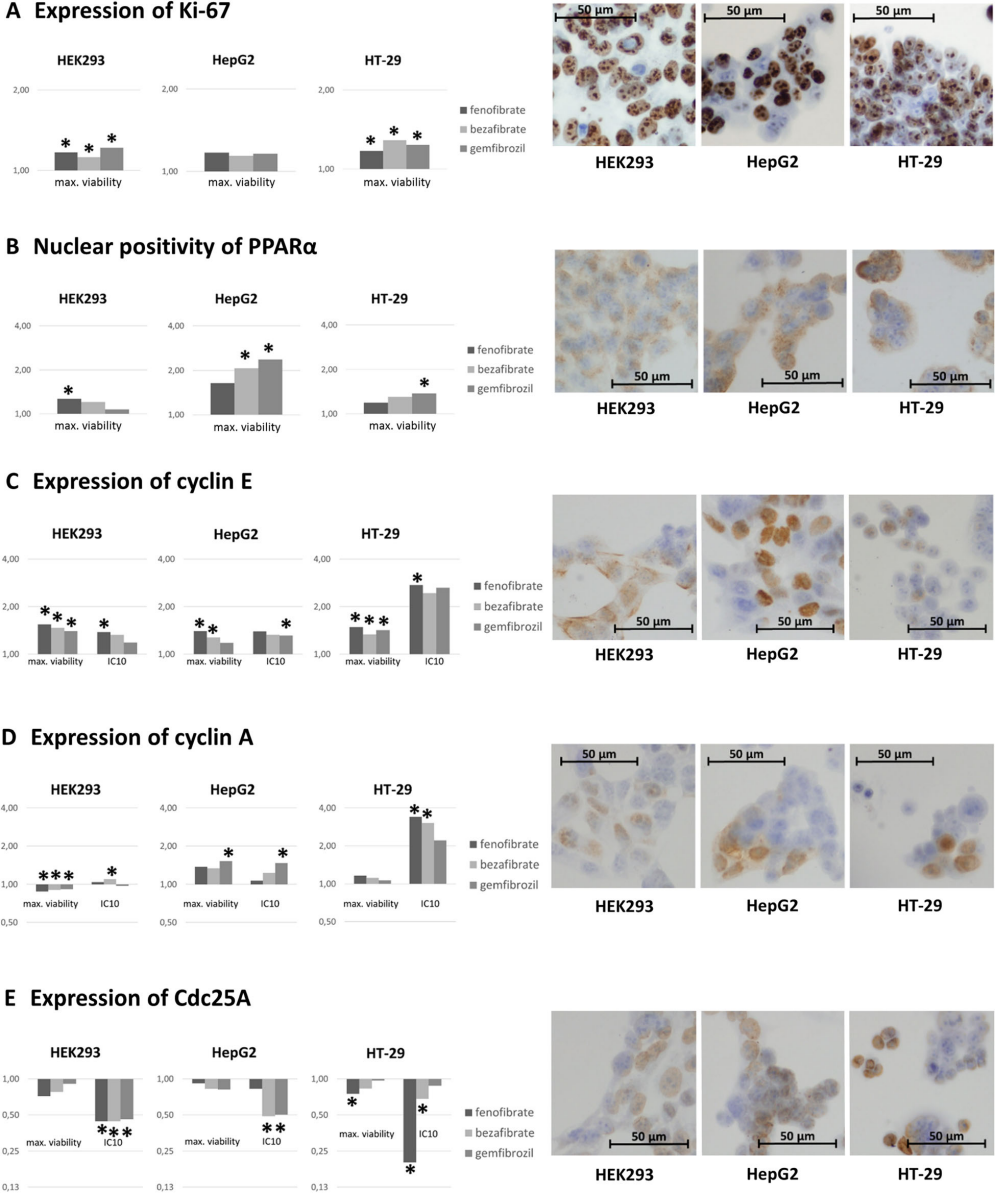
Changes in expression of Ki-67, subcellular localization of PPARα and expression of cell cycle regulators. **a** Ki-67 is a marker of cell proliferation which is independent on specific phase of cell cycle (G1, S, G2, M). Increased number of Ki-67 positive cells was detected after treatment at maximal viability concentrations of used fibrates. These results confirm increased viability after fibrate treatment determined by WST-1 test. **b** In all three tested cell lines, we detected an increased number of cells with nuclear positivity of PPARα after fibrates treatment. Both, cytplasmic and nuclear positivity, is apparent. **c** Expression of cyclin E is increased in HEK293, HepG2, and HT-29 cell lines after fibrate treatment. **d** Expression of cyclin A is increased in carcinoma cell lines HepG2, and HT-29. In HEK293 cell line, there is slight decrease in cells treated by maximal viability concentrations of fibrates. **e** Expression of Cdc25A is decreased in HEK293, HepG2, and HT-29 cell lines after fibrate treatment. Graphs display results as fold changes. * Statistically different from control value at *p* < 0.05. Microphotographs (magn. 400×) show expression of proteins of interest in control cells (treated by 0.1 % DMSO)

To confirm increased proliferation after the fenofibrate, bezafibrate, and gemfibrozil treatment, we used immunocytochemical detection of proliferation marker Ki-67. Ki-67 is a nuclear protein associated with cellular proliferation and it is expressed independently on specific phase of cell cycle (G1, S, G2, M). All cell lines were treated by maximal viability concentrations of fibrates determined by WST-1 test. Ratio of Ki-67 positive cells were increased after fibrates treatment (Table 2 and Fig. 2, part A) as fold change. These result confirmed increased proliferation detected by WST-1 test.

**Table 2:** Ratio of Ki-67, cyclin E, cyclin A, and Cdc25A positive cells in tested cell lines obtained by immunocytochemistry

	antigen	Ki-67	cyclin E		cyclin A		Cdc25A	
	treatment	max. viability (% of positive cells)	max. viability (% of positive cells)	IC10 (% of positive cells)	max. viability (% of positive cells)	IC10 (% of positive cells)	max. viability (% of positive cells)	IC10 (% of positive cells)
HEK293	control	73.44 ± 6.39	56.82 ± 9.68		55.19 ± 4.00		57.25 ± 9.14	
	fenofibrate	95.04 ± 7.49	82.11 ± 3.58	73.39 ± 9.26	44.99 ± 0.62	53.49 ± 5.40	37.04 ± 7.65	22.82 ± 8.84
	bezafibrate	91.31 ± 6.83	77.95 ± 2.39	70.33 ± 8.29	46.35 ± 0.05	56.41 ± 0,42	40.23 ± 4.80	22.79 ± 10.74
	gemfibrozil	98.82 ± 0.45	74.42 ± 2.46	62.89 ± 2.15	46.98 ± 2.30	49.83 ± 5.31	47.03 ± 2.94	23.79 ± 7.75
HepG2	control	77.59 ± 1.81	46.65 ± 7.69		34.22 ± 6.06		48.70 ± 6.71	
	fenofibrate	83.14 ± 7.05	65.67 ± 3.35	65.21 ± 29.03	42.55 ± 2.23	33.08 ± 3.49	44.92 ± 5.65	40.56 ± 6.81
	bezafibrate	80.82 ± 5.77	59.60 ± 2.33	62.2 ± 8.86	41.59 ± 0.23	38.09 ± 12.48	40.45 ± 5.12	23.92 ± 7.96
	gemfibrozil	82.39 ± 5.54	55.08 ± 2.31	61.57 ± 3.08	47.04 ± 3.72	45.43 ± 4.03	39.94 ± 9.19	24.63 ± 7.98
HT-29	control	72.83 ± 4.33	15.69 ± 1.39		18.39 ± 2.82		57.66 ± 9.41	
	fenofibrate	85.96 ± 5.09	24.07 ± 1.02	44.34 ± 6.62	20.36 ± 2.53	59.45 ± 13.84	39.53 ± 4.00	10.60 ± 9.70
	bezafibrate	94.37 ± 1.07	21.64 ± 2.04	39.38 ± 12.53	19.53 ± 1.32	53.23 ± 6.14	43.77 ± 8.44	35.88 ± 8.40
	gemfibrozil	90.59 ± 0.88	23.02 ± 2.25	42.64 ± 11.07	18.65 ± 1.13	38.70 ± 21.33	51.13 ± 6.97	46.26 ± 4.32

We evaluated % of positive cells in 3 different fields of vision for these antigens in control and fibrates treated HEK293, HepG2, and HT-29 cell lines. Cells were treated by maximal viability and IC10 concentrations of used fibrates. Results are shown as % of positive cells ± SD (*n* = 3)

### Changes in subcellular localization of PPARα

To confirm that an increase in proliferation and changes in expression of cell cycle regulators could be PPARα-dependent, we investigated presence and subcellular distribution of PPARα. We detected both, cytoplasmic and nuclear localization of PPARα. In all three tested cell lines we detected an increased number of cells with nuclear positivity of PPARα in comparison to control cells. The results are shown in Fig. 2, part B as fold change.

### Changes in ratio of cells expressing cell cycle regulation proteins

To investigate why cell proliferation is increased after the treatment with fibrates, we used immunocytochemistry for detection of cell cycle regulation protein expression, namely cyclin E, cyclin A, Cdc25A in control cells (treated by 0.1 % DMSO) and cells treated by maximal viability concentration and IC10 of fibrates determinated by WST-1. The expression of all tested proteins was detected in all tested cell lines.

Cyclin E, cyclin A, and Cdc25A are regulators of late G1 and S phase of the cell cycle. Results for all tested cell lines are summarized in Table 2. Changes in expression of theese proteins are shown in Fig. 2, part C, D, E as fold change. Statistically significant changes are labed by *, for *P* values, see Additional file 4. Briefly, increased number of cells expressing cyclin E in all tested cll lines was detected. Moreover, number of cells expressing cyclin A was increased in carcinoma cell lines (HepG2, HT-29). Cdc25A is downregulated in all tested cell lines. All these changes are concentration-dependent.

### Confirmation of p53 presence

**Fig. 3:**
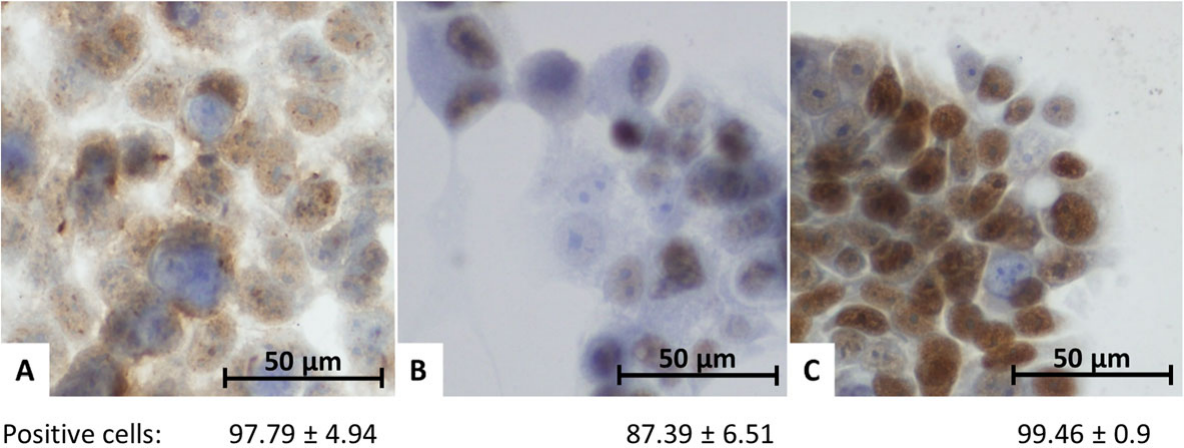
Expression of p53 in HEK293 (**a**), HepG2 (**b**), and HT-29 (**c**) cell lines. In all tested cell lines, he majority of cells was positive for p53. The p53 protein was predominantly nuclear, cytoplasmic expression was also detected (magn. 400×). Ratio of positive cells is displayed as average ± SD

We also confirmed presence of p53 in all tested cell lines. In all three tested cell lines, the majority of cells were positive for this protein. We detected both, cytoplasmic and nuclear positivity. Results of immunohistochemistry staining and ratio of positive cells (displayed as average ± SD) after treatment by 0.1 % DMSO are shown in Fig. 3.

**Fig. 4:**
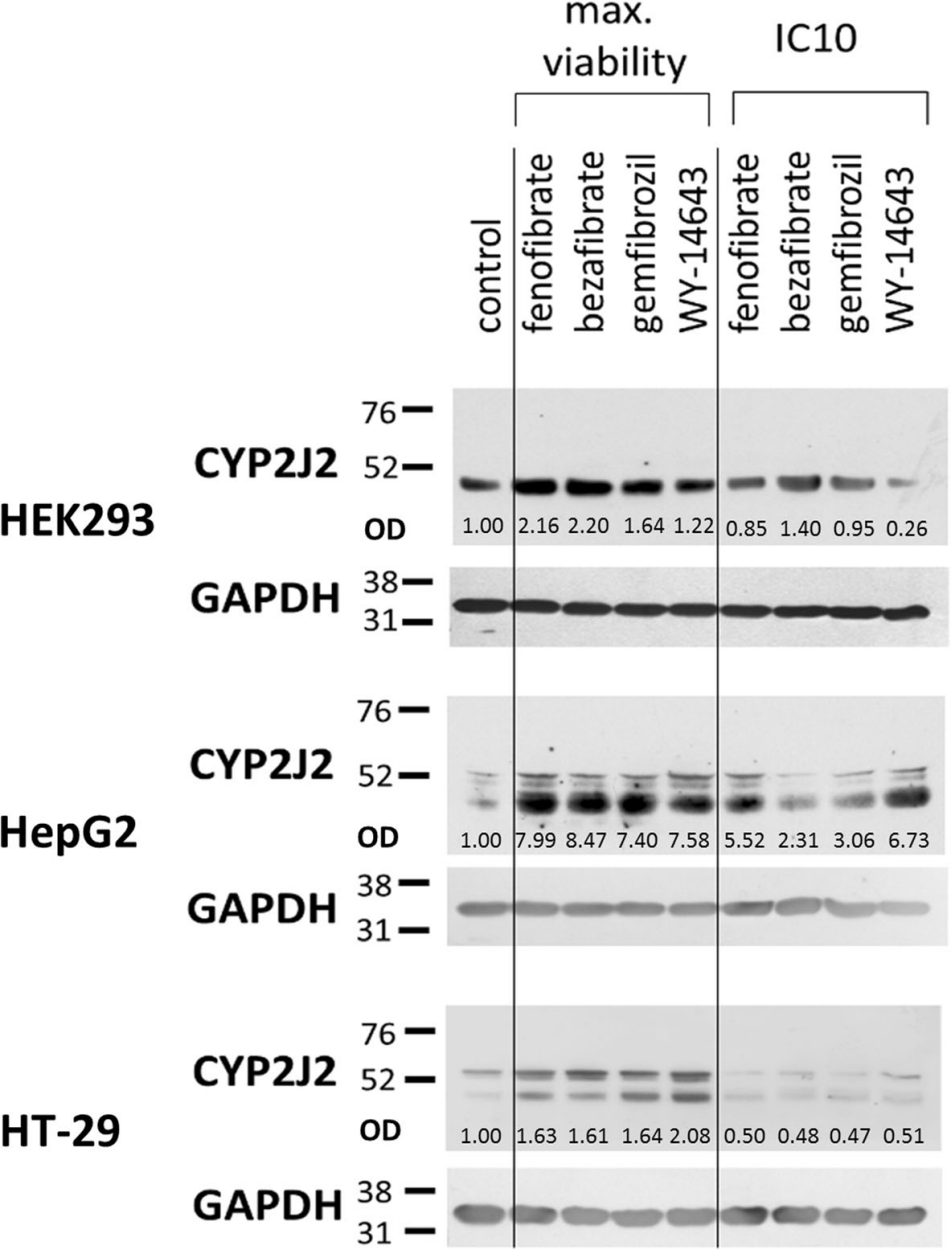
Expression of CYP2J2 in HEK293, HepG2, and HT-29 cell lines in control cells and after fibrates treatment in concentration which promotes viability and IC10. Generally, in maximal viability concentrations, CYP2J2 protein expression is elevated in all tested cell lines. In IC10 concentrations, CYP2J2 is returned to control levels or slightly downregulated. The higher expression of CYP2J2 could explain increase in viability of the cells. Detection of GAPDH expression was used as endogenous control. Relative protein expression was evaluated by measuring optical density (OD)

### Western blot analysis of CYP2J2 expression

We hypothetized if observed changes in cell viability are connected with changes of expression of CYP2J2. CYP2J2 were detected in all tested cell lines. We detected obvious increase in CYP2J2 expression after treatment in proliferation concentrations. The cells treated with IC10 concentrations showed return to CYP2J2 expression to level comparable to control cells or slight downregulation. Only one exception is remaining of higher expression of CYP2J2 in HepG2 cell line after WY-14643 treatment at IC10 concentration. Representative results are shown in Fig. 4.

### Cell cycle analysis

**Fig. 5:**
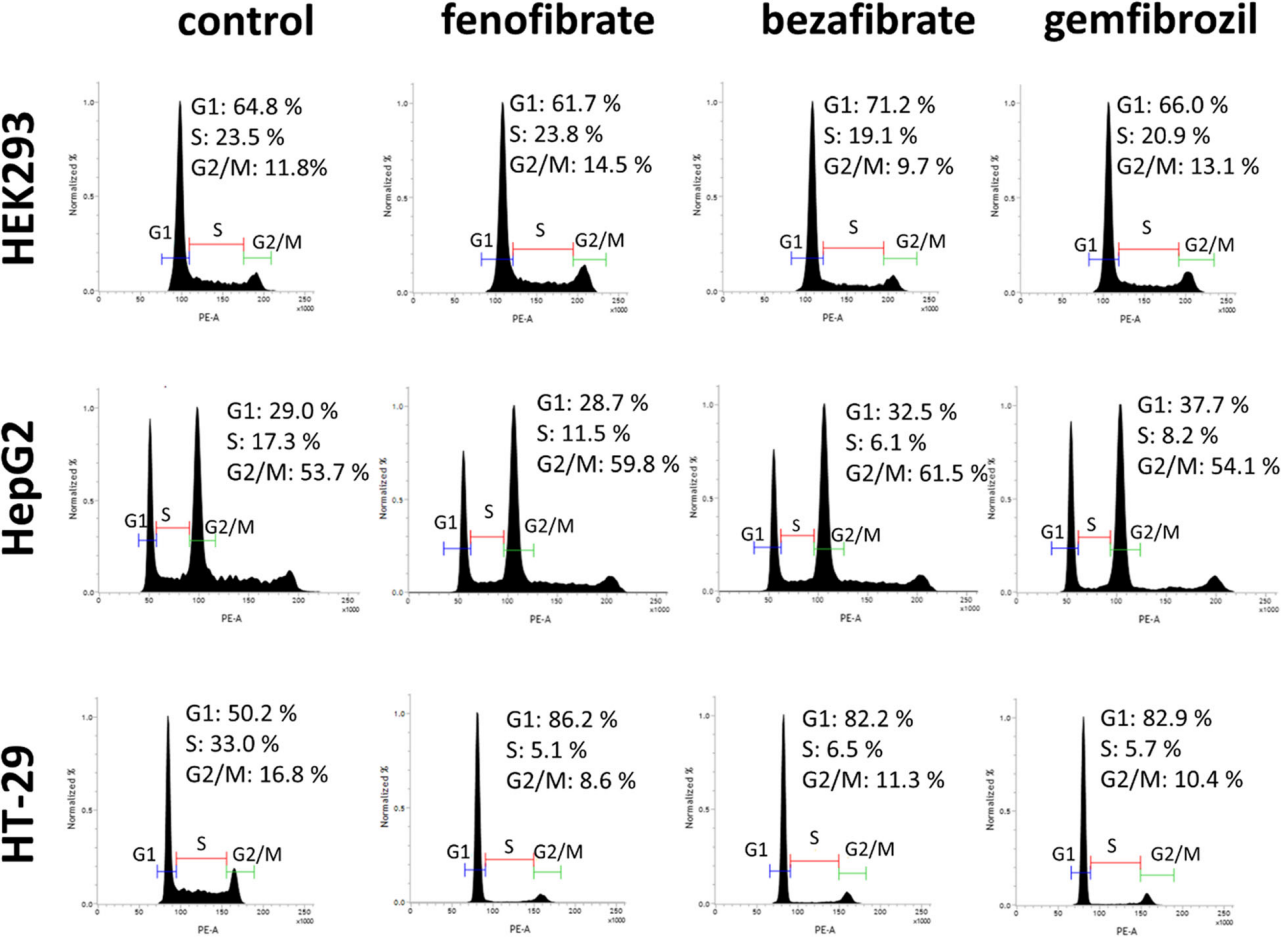
Results of cell cycle analysis. There is an accumulation of cells in G1 phase after fibrate treatment in IC10 concentration

We detected no changes in distribution of cell cycle phases after maximal viability treatment in all tested cell lines in comparison to control cells (treated by 0.1 % DMSO) for all three tested cell lines. Cells treated by IC10 concentrations of tested fibrates showed an increase of cells in G1 phase in comparison to control cells (see Fig. 5). We also detected an increased number of tetraploid cell in HepG2 cell line after the gemfibrozil treatment.

## Discussion

PPARα is a ligand-activated transcription factor involved in regulation of lipid and energy metabolism, inflammation, and xenobiotic metabolism. There are many of both, exogenous and endogenous compounds which serve as PPARα ligands. PPARα ligands include fibrates, phtalates, herbicides, saturated and unsaturated fatty acids, prostaglandins, leucotriene B4, epoxyeicosatrienoic acids (EETs), and etc. [3].

PPARα ligands such as fenofibrate, bezafibrate, and gemfibrozil are well kown hypolypidemic drugs and thus, they can improve clinical consequences of metabolic disorders asocciated with increased cancer risk. They have long history of clinical usage, been shown to be well tolerated and to have limited side effects and/or toxicity. Long-term administration of PPARα agonists causes liver cancer in rodents. However, this effect is not evident in humans [1]. Moreover, they have been considered as potential anticancer agents [5–9].

In this study, we investigated effects of a wide range of concentrations of fenofibrate, bezafibrate, gemfibrozil, and WY-14643 on viability of three human cell lines: HEK293, HepG2, and HT-29. Our results shown the same trend in all estimated cell lines. At lower concentration of all tested fibrates, increased proliferation is observed, whereas at higher concentration, repression is apparent. Increased viability of all cell lines treated by lower concentrations of fibrates was comfirmed by increased Ki-67 expression in tested cells. Fenofibrate, bezafibrate, and gemfibrozil are widely used for dyslipidemia treatment. Unfortunately, with exception of HEK293 cells treated by fenofibrate,concentrations of fibrates with a promotive effect for proliferation are in the range of concentrations which are reached in patient plasma after normal therapeutical dosing of these drugs. Our data are with good agreement with Suchanek et al. They detected increased proliferation after WY-14643 and clofibrate treatment in human breast cancer cell lines. The increase in proliferation was more significant in lower concentration of WY-14643, wherease higher concentration produced only a slight increase in proliferation of tested cell lines [12].

It has been described a lot of effects of PPARα ligands on cancer for cancer [4, 10, 13–15, 17]. Animal experiments provide promising results in field of PPARα-dependent regulation of CYP epoxygenases and consequent marked reduction of tumour mass volume and vascularization in mice [17]. CYP epoxygenases (mainly CYP2C and CYP2J subfamilies) convert arachidonic acid (AA) to four regioisomers of EETs. AA is dietary polyunsaturated ω-6 fatty acid which is part of normal nutrition. EETs are involved in inflammation, mitogenesis, cell signalling, angiogenesis, regulation of vascular tone, and ion channels. It has been described that EETs promote tumour growth by increasing cell proliferation and apoptosis inhibition [16, 18]. CYP epoxygenases are significantly expressed in variety of tumour tissues. These enzymes and their metabolites are possibly related to the incidence and progression of tumours [18–20].

In mice, it has been showen that PPARα agonists downregulate CYP epoxygenases expression. On the other hand, expression of human CYP2C and CYP2J mRNAs are upregulated by PPARα ligands [4]. Although the downregulation of CYP2C is apparent also in PPARα-humanized mice, differences in regulation of CYP2C in mice and humans could be attributed to differences in the regulatory regions of the responsive genes, which have been described for several genes before [21, 22]. This inter-species differences in regulation of CYP2C proteins expression should be considered in evaluation and transfer of rodent study results to humans.

Our results show that fibrates affect CYP2J2 expression in dose-dependent manner. CYP2J2 level is increased in tested cell lines after lower-concentrations fibrates treatment. This increase is probably followed by increase of cytoprotective EETs concentration in tested cells and could explain increased viability of the cells.

To support our assumption that these efects are PPARα-dependent, we estimated presence and subcellular localization of PPARα in tested cell lines in control and fibrate treated cells. While localization of PPARα is widely regarded as nuclear, cytoplasmic localization has also been described [23–25]. We detected both, nuclear and cytoplasmic localization of PPARα. Cells with nuclear positivity of PPARα were accumulated after PPARα ligands treatment. Recently, Umemoto et Fujiki [26] have described dynamic shuttle between nucleus and cytoplasm which is regulated by multiple pathways and nuclear transport of PPARα is accelerated by its ligands which is in good agreement with our results.

In further experiments, we investigated expression of cell cycle regulatory proteins after fibrate treatment. Cell cycle progression is mainly controlled by cyclins and cyclin-dependent kinases (CDKs). Activities of cyclin/CDKs complexes are regulated on different levels, such as transcription of their genes, proteolysis, subcellular localization, binding of specific inhibitors, and reversible phosphorylation [27]. Common feature of all tested fibrates are changes in expression of Cdc25A and cyclin E in all three tested cell lines and cyclin A in carcinoma-derivated cell lines HepG2 and HT-29. These effects are concentration-dependent. Cyclin E and cyclin A are regulators of late G1 and S phases of cell cycle. Both cyclins make complex with cyclin-dependent kinase 2 (CDK2). For activation of these complexes, dephosphorylation of CDK2 by Cdc25A is needed. All together, these proteins induce progression from G1 to S phase of cell cycle. Overexpression of Cdc25A phosphatase is often observed in cancer and results in poor prognosis. Downregulation of Cdc25A could play a role in the prevention of uncontrolled cell growth as long as p53 is intact [27]. In maximal viability concentrations, Cdc25A downregulation seems to be insufficient for cell cycle arresting and together with upregulation of cyclin E (and cyclin A), fibrates promote cell proliferation. Sufficient downregulation of Cdc25A for arrest of cell cycle is probably reached in IC10.

## Conclusion

According to information mentioned above, it seems that PPARα is not pure oncogene or tumour suppressor. Althought many of anticancer effects of fibrates are described in literature, based on our research, we suggested there is no anti-cancer effect of fibrates in tested carcinoma cell lines. The increased proliferation of the cells are accompanied by the induction of CYP2J2 protein which could explain this phenomenon. Because of widen clinical utilization of fibrates, their effect on cell viability needs further investigation and their usage should be considered carefully.

## Additional files

Additional file 1: Summary of possible role of PPARα in cancer according to literature [3, 4, 10, 13–16]. (TIF 1675 kb) Additional file 2: Relative viability of cells treated by two different concentrations of DMSO. The viability of cells treated by 0.1 and 1 % DMSO were compared to untreated cells growth in standart condition. There is no significant effect (*t*-test, *p* < 0.05) of DMSO on cell viability. (TIF 93 kb) Additional file 3:
*P* values obtained for changes in cell viability after fibrates treatment in plasma concentration ranges. Statistically different from control value are red (*t*-test, *p* < 0.05). (XLSX 11 kb) Additional file 4:
*P* values obtained for changes in expression of Ki-67, cyclin E, cyclin A, Cdc25A and subcellular localization of PPARα receptor. Statistically different from control value are red (*t*-test, *p* < 0.05). (XLSX 9 kb)

## Abbreviations

FBS: Fetal bovine serum
HAT: Histon acetyl transferase
HDAC: Histon deacetylase
PPAR: Peroxisome proliferator-activated receptor
PPRE: Peroxisome proliferator response element
RXR: Retionoid X receptor
STR: Short tandem repeat
