# Supplementary figures and images for: Stimulating effect of normal-dosing of fibrates on cell proliferation: word of warning
Source: Lipids Health Dis. 2016 Sep 22;15:164. doi: 10.1186/s12944-016-0335-z (PMC5034623; doi:10.1186/s12944-016-0335-z)

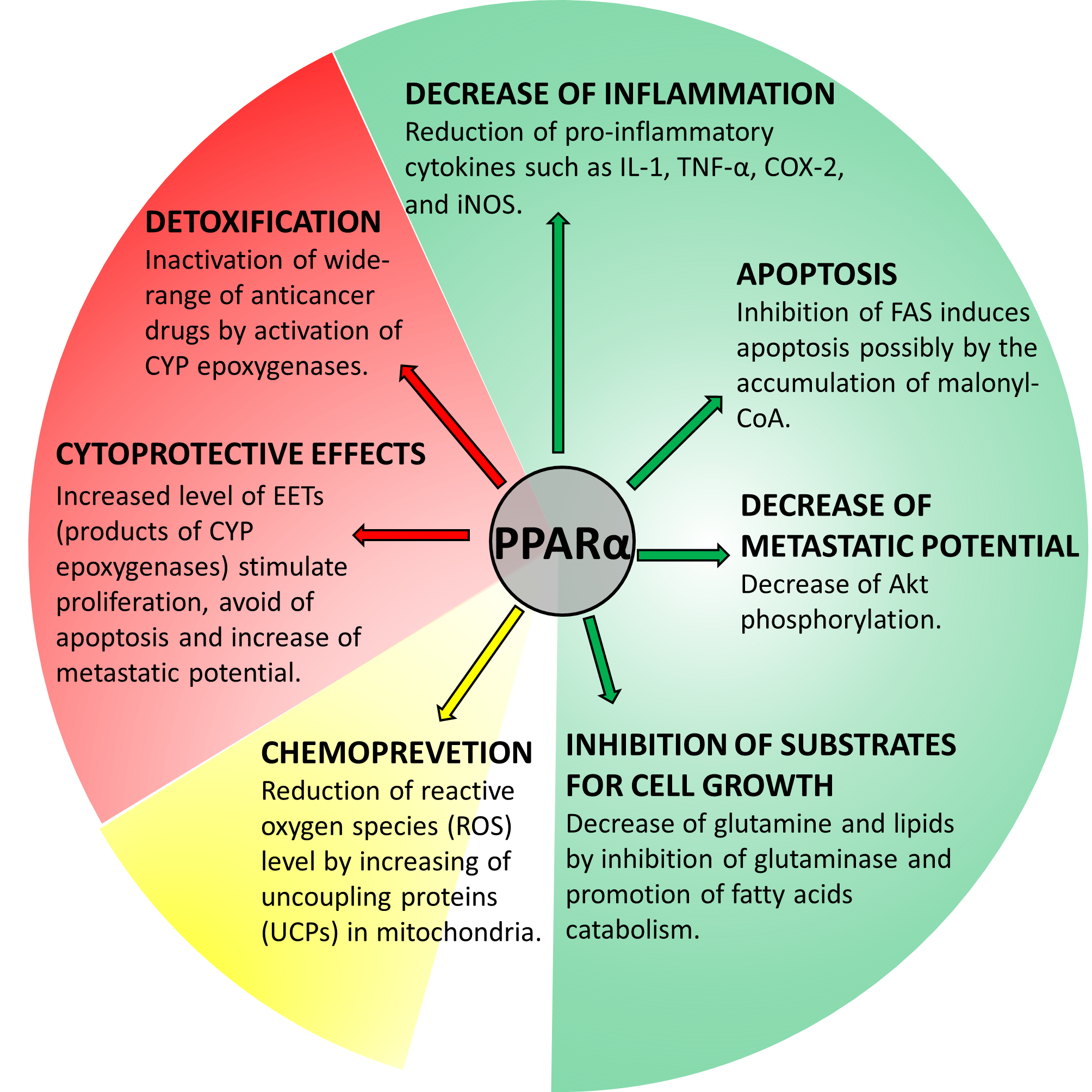

Supplement: Additional file 1: — Summary of possible role of PPARα in cancer according to literature [3, 4, 10, 13–16]. (TIF 1675 kb) [file 12944_2016_335_MOESM1_ESM.tif]

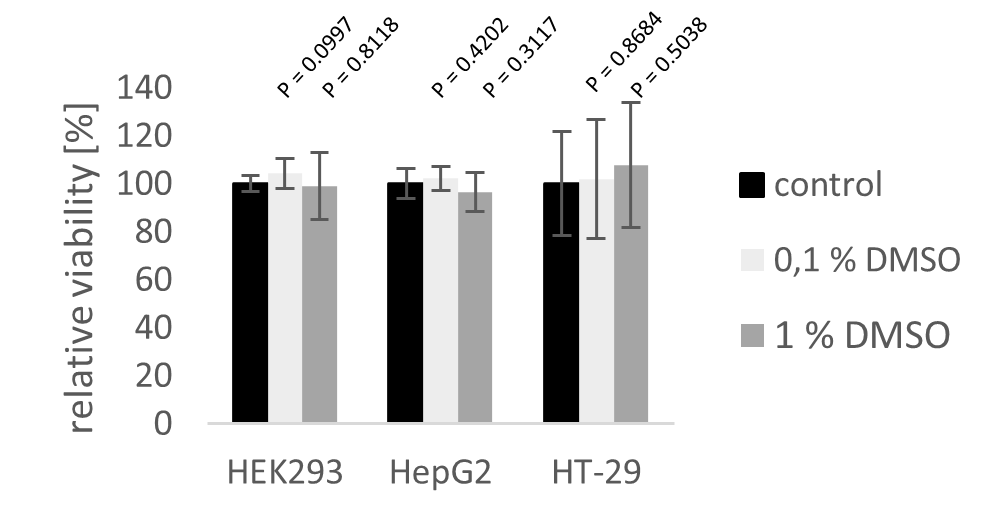

Supplement: Additional file 2: — Relative viability of cells treated by two different concentrations of DMSO. The viability of cells treated by 0.1 and 1 % DMSO were compared to untreated cells growth in standart condition. There is no significant effect (t-test, p < 0.05) of DMSO on cell viability. (TIF 93 kb) [file 12944_2016_335_MOESM2_ESM.tif]
